# 

**Published:** 1892-04-09

**Authors:** 


**Notices of Births, Deaths, and Marriages,** are inserted in this column at a charge of 2s. 6d. for an announcement not exceeding four lines.

### NOTICE TO CORRESPONDENTS.

All MS., Letters, Books for Review, and other matters intended for the Editor should be addressed The Editor, The Lodge, Porchester Square, London, W.

The Editor cannot undertake to return rejected M.S., even when accompanied by stamped directed envelope.

### Notice to Advertisers and Subscribers.

All Advertisements, Orders for Copies of The Hospital, and Business Communications should be addressed to The Publisher, *not to the Editor*, The Hospital, 140, Strand, W.C.

The Cost of Subscription, post free, is as follows:—Per week, 2½d.; per quarter, 2s. 6d.; per half-year, 5s.; per annum, 8s. 6d. Foreign:—Per annum, 10s. 8d.; payable, in all cases, in advance.

**"Burdett's Hospital Annual," 1891—1892.**

Third year of publication. 600 pp. Boards, gilt lettering. A most complete guide to British and Colonial Hospitals, Dispensaries, Nursing and Convalescent Institutions, and Asylums. Price 3s. 6d. Published by The Scientific Press (Limited), 140, Strand, W.C.

**NOTICE.—The Index for Vol. X. (ending September, 1891), is on sale at the Office of this Journal, price 2½d., post free.**

### Scraps and Gleanings.

The new out-patients' department of the Manchester Consumption Hospital was opened on the 30th inst. by Miss Balfour.

Mr. Oliver Heywood, the well-known banker of Manchester, who died last week, has left £2,000 to the Manchester Infirmary.

A subscription ball in aid of the Miller Hospital and Royal Kent Dispensary will take place at the Ship Hotel, Greenwich, on Wednesday, April 27th.

The dances given in aid of the North-West London Hospital proved a great success. After paying all expenses, the sum of £491 was handed over to the hospital.

A rose show and fruit and flower fête in aid of the Royal Hospital for Women and Children will be held by the Lady Mayoress at the Mansion House on June 24th.

Mr. Harry Furniss, who has been ordered by his medical adviser to take a sea voyage, has left England for the United States, and will be absent about six weeks.

Princess Louise (Marchioness of Lorne) has consented to open, some time in May, the new Convalescent Home in connection with the Victoria Hospital for Children at Chelsea.

Under the will of the late Sir Charles John Wingfield, more than sixty London hospitals and philanthropic institutions receive £100 each, and about thirty others receive £50 each.

The annual report of the Surrey County Hospital states that the number of in-patients last year was 843, against 774 in 1890. The total expenditure exceeded the income by £145 3s. 5d.

At a meeting yesterday of the Governors of the Foundling Hospital Mr. Robert Grey was unanimously elected Treasurer of the institution, in succession to the late Mr. George Burrow Gregory.

A concert was given by the members of the Whitley Choral Society last week in the dining hall of the Whitley Convalescent Home to the patients, nurses, and other members of the institution.

An anonymous donor has given £1,000 towards the enlargement of the Royal Alexandra Hospital at Rhyl. The Duke of Westminster and Mr. Barnford Heckett have also contributed large sums.

During the fifteen years that St. John's Convalescent Home, Southsea, has been instituted 1,213 men and boys have passed through the home. Of these 665 left cured, and 463 improved in health.

The Bishop of London recently visited St. Mary's Hospital and administered the rite of confirmation, in the hospital chapel, to a boy, who is a patient in the children's ward, suffering from heart disease.

As a suitable way of commemorating the jubilee of the Brompton Hospital, the Committee have decided to enlarge the chapel. A gift of £1,000 towards the fund has been received from a friend. A sum of over £2,000 is still needed.

At the second annual meeting of the Wolverhampton and District Hospital for Women and Children, the balance-sheet showed receipts £394 2s. 11½d., and expenses £426 5s. 8d. Total number of out-patients was 1,916, and in-patients 84.

At the annual meeting of the Governors of the Leamington Provident Dispensary it was stated that the receipts amounted to £1,450 8s. 7d., and the expenditure to £1,071 18s. 9d., leaving a balance in the Treasurer's hands of £378 4s. 10d.

Princess Louise (Marchioness of Lorne) will distribute the St. John Ambulance Association certificates to officials of the medical mission smacks of the Mission to Deep Sea Fishermen, at that society's annual meeting at Exeter Hall, on May 16th.

Mr. Richard Cadbury has handed over to the Mayor of Birmingham his late residence, Moseley Hall, which he has partially endowed as a convalescent home for children. The institution has accommodation for 150 beds, and, with the endowment, is worth £30,000.

At the quarterly meeting of the Board of Delegates to the Hospital Saturday Fund, the Workshops and Streets Collection Committee reported that 1,497 new and 804 old firms had been visited since the last report, 711 sheets and boxes issued and 484 new firms registered.

At Easter a ball under distinguished patronage will be held at Brighton in aid of the Hospital for Women and Children, West Street. This year the ball promises to be even a greater success than usual. The Mayor (Alderman D. Ewart) is Vice-President of the institution.

The late Baroness Bettina Rothschild and her husband gave largely to all charities, but more especially to those connected with children. Besides contributing handsomely to asylums and children's hospitals in Vienna the Baroness founded hospitals and asylums on her property in Gaming and in Waidhofen.

Mozart's watch, originally presented to the composer by the Empress Maria Theresa, has just been deposited in the Mozarteum at Salzburg. It is, of course, an old-fashioned but very handsome affair, the case being set with diamonds of considerable value, and it was formerly the property of Herr Pfeffer, of Buda Pesth.

The Children's Fancy Dress Ball, postponed in January on account of the death of the Duke of Clarence, has been fixed for Thursday, April 21st, at Princes' Hall. The object is the endowing of a cot in the Victoria Hospital for Children, which the Princess of Wales has named after her late son. The Children's Salon has received the patronage of six members of the Royal family.

At a special meeting of the committee of the Richmond Hospital, Surrey, held last autumn, it was resolved that, owing to the insufficient accommodation for the nursing staff, and the need of a special ward for children, to open a new building fund, and that all legacies, unless otherwise specified, be paid into it.

The Men's Convalescent Home, which was established last year on the Mendips by Colonel Hill, M.P., and Miss Mabel Hill, was reopened last week for the summer season. "Cosy Corner," as the home is called, is situated in a pretty sheltered spot nine hundred feet above the sea level. It is intended solely for convalescents or persons in a weak state of health requiring change of air, rest, and good food.

### NOTICE.

### FURNITURE AND APPLIANCES.

We purpose giving a descriptive account with illustrations of all the new appliances and furniture used in hospitals and asylums, and we desire to secure the co-operation and the superintendence of the medical staff, secretaries, and matrons, so that the whole ground may be covered by this section, which should prove very interesting and helpful in many ways. Each institution which has any fittings or furniture which are considered specially suitable or novel are invited to send a description with sketch for publication in this column. All communications should be marked "Furniture and Appliances," and addressed to the Editor of The Hospital, 140, Strand, W.C.

### THE STUDENT OF MEDICINE.

[All communications for this Supplement should be addressed to The Editor of The Hospital, at 140, Strand, with the words, "Students' Column," written in the left hand top corner of the envelope.]

### APPOINTMENTS.

Ernest Horsford Bingley, L.S.A.Lond., appointed House-Surgeon to the Chichester Infirmary. Wm. Charnley, M.D. Cantab., M.R.C.S., appointed Honorary Oculist to the South Shropshire Infirmary. Thomas Hugh Dickson, M.A., M.B., B.C.Cantab., M.R.C.S.Eng., L.R.C.P.Lond., appointed Medical Inspector H.M. Customs. Dundas Grant, M.A., M.D., F.R.C.S. Eng., appointed Surgeon for the Throat and Ear to the West End Hospital for Nervous Diseases. George Hern, M.R.C.S., L.R.C.P., L.D.S.Eng., appointed Demonstrator to the Dental Hospital of London, Leicester Square. Charles Christopher Heywood, M.A., M.B.Cantab., M.R.C.S., appointed House Surgeon to the Salford Royal Hospital. A. R. Rocke Hudson, L.R.C.P., L.R.C.S.I., Springfield, Widnes, District Medical Officer to Wesleyan and General Assurance Company. G. B. Jameson, M.B., C.M.Edin., appointed House-Surgeon to the Central London Ophthalmic Hospital, Gray's Inn Road, W.C. Frederick Knight, M.D.Lond., appointed Physician to the Swansea Hospital. F. P. Lansdown, M.R.C.S., reappointed Senior Surgeon to the Bristol General Hospital. John Fletcher Little, M.B.Camb., M.R.C.P.Lond., appointed Assistant Physician to the London Temperance Hospital. Archibald Mackintosh, M.B.Edin. and C.M., appointed House-Physician to the Leith Hospital. Y. Hubert Mills, F.R.C.S., M.B., appointed Surgical Registrar to the London Hospital, Whitechapel. J. T. J. Morrison, M.A., B.C.Cantab., F.R.C.S.Eng., appointed Central Divisional Surgeon to the Birmingham Police Force and Fire Brigade. Gervase Newby, M.R.C.S.Eng., L.R.C.P.Lond., appointed Junior House-Surgeon to the Salford Royal Hospital. Benjamin Ward Richardson, M.A., M.D.St.And., F.R.C.P. Lond., F.R.S., appointed Physician to the London Temperance Hospital. T. E. Spencer, M.R.C.S., L.R.C.P., appointed House-Surgeon to the Weston-super-Mare Hospital. E. Mansel Sympson, M.D., B.C.Cantab., appointed Surgeon to the Lincoln County Hospital. R. H. Tompsett, L.R.C.P.Lond., M.R.C.S., appointed Second Assistant Medical Officer to the Fulham Road Workhouse and the Infirmary, St. George's Union.

### VACANCIES.

### Resident Medical Officers.

The following vacancies are announced this week, the amount of salary in each case being given after the name of the institution :

Derbyshire Royal Infirmary, Resident Assistant House Surgeon. Appointment for six months, but eligible for an additional six months—£10 for first six months, £25 for second six months, with board, &c.

Londonderry Lunatic Asylum, Assistant Medical Officer—Salary £100 per annum, &c.

Manchester Southern and Maternity Hospital House-Surgeon.

Manchester Southern and Maternity Hospital, House-Surgeon—Honorarium at the rate of £75 per annum.

Metropolitan Hospital, Kingsland Road, N.E., House-Physician. Must be M.R.C.S.Eng. Appointment for six months—Salary at the rate of £60 per annum, &c.

Metropolitan Hospital, Kingsland Road, N.E., House-Surgeon. Must be M.R.C.S.Eng. Appointment for six months—Salary at the rate of £60 per annum, with board, &c.

Metropolitan Hospital, Kingsland Road, N.E., Assistant House-Surgeon. Must be M.R.C.S.Eng.—Board, &c.

Nottingham Borough Asylum, Mapperley Hill, Nottingham, Assistant Medical Officer, unmarried—Salary, £125 per annum, &c.

Paddington Green Children's Hospital, W., House-Surgeon. Appointment for six months—Salary at the rate of £55 5s. per annum, with Board, &c.

Royal Free Hospital, Gray's Inn Road, Senior Medical Officer, doubly qualified—Salary £100 per annum, with Board, &c.

Royal Free Hospital, Gray's Inn Road, Junior Medical Officer—Board, &c.

### Non-Resident Medical Officers.

College of State Medicine, Research Scholarship, tenable for one year—Salary £100.

Haverstock Hill and Malden Road Provident Dispensary, 132, Malden Road, N.W., Medical Officer.

Manchester Royal Infirmary, Dispensary, and Lunatic Hospital or Asylum, Honorary Assistant-Physician.

Victoria Hospital for Children, Queen's Road, Chelsea, Honorary Medical Officer to New Convalescent Branch at Broadstairs to be opened in May.

---

Southall-Norwood Local Board, Analyist.

### THE STUDENT OF MEDICINE—(*continued*).

### NOTES FROM THE SCHOOLS.

**University of Durham School of Medicine, Newcastle.**

On Thursday, 17th ult., the last of the students' assemblies was held at the New Assembly Rooms, Barras Bridge. The guests were received by Mrs. Neesham and Mrs. Bedson. About 150 were present, and the dance was a most enjoyable one.

The following evening, Friday, the 13th, was the occasion of the annual dress concert of the Musical Society. This was held in the large library of the College of Medicine, under the presidency of the President of the Society, Dr. Howden, who was ably supported by the Hon. Secretaries, Messrs. Peacock and Hodgson. The concert was most ably organised and as efficiently carried out. Especial reference must be made to the violin playing of Miss Florence Shaw, which was most pleasing in execution and artistic feeling. Miss Ada Davis and Miss Kate Neesham shared the vocal honours, which they richly deserved. After the concert the room was cleared for a dance, by the kind permission of Dr. Philipson, the newly-elected President of the College of Medicine, which post had just become vacant by the death of Dr. Heath.

**Royal College of Surgeons.**

FEES FOR THE MEMBERSHIP: NEW SYNOPSIS IN BIOLOGY.—The Committee of Management on the arrangement of fees under the five years' curriculum have recommended that the fee for each of the first three examinations should be 10 guineas, and for the final examination 5 guineas, and that the total amount of fees for re-examination should be raised from 28 to 30 guineas. The Committee have also reported on the subjects of the first and second conjoint examinations under the regulations of the five years' curriculum. The chief item of the report was the synopsis of Elementary Biology, as follows: 1. Chemical composition and properties of protoplasm; nature of cells; cell division; outlines of cell structure of chief tissues of plants and animals; fertilisation and the early stages of development. 2. Unicellular organisms; outlines of organisation and life-history of amœba, gregarina, vorticella, yeast plant, bacteria, protococcus, and glœocapsa. 3. Chief differences between plants and animals. 4. Anatomy, life-history, and embryology of hydra. 5. Anatomy and life-history of the leech, liver fluke, tapeworms, and round worms. 6. General and distinctive characters of vertebrate animals. 7. Comparison of man's structure with that of other mammalia.

